# Supplementary material for: Human spinal cord activation during filling and emptying of the bladder
Source: Nat Commun. 2025 Jul 15;16:6506. doi: 10.1038/s41467-025-61470-1 (PMC12264007; doi:10.1038/s41467-025-61470-1)
Supplement: Supplementary file 1 — Supplementary Information [file 41467_2025_61470_MOESM1_ESM.pdf]

Supplementary materials

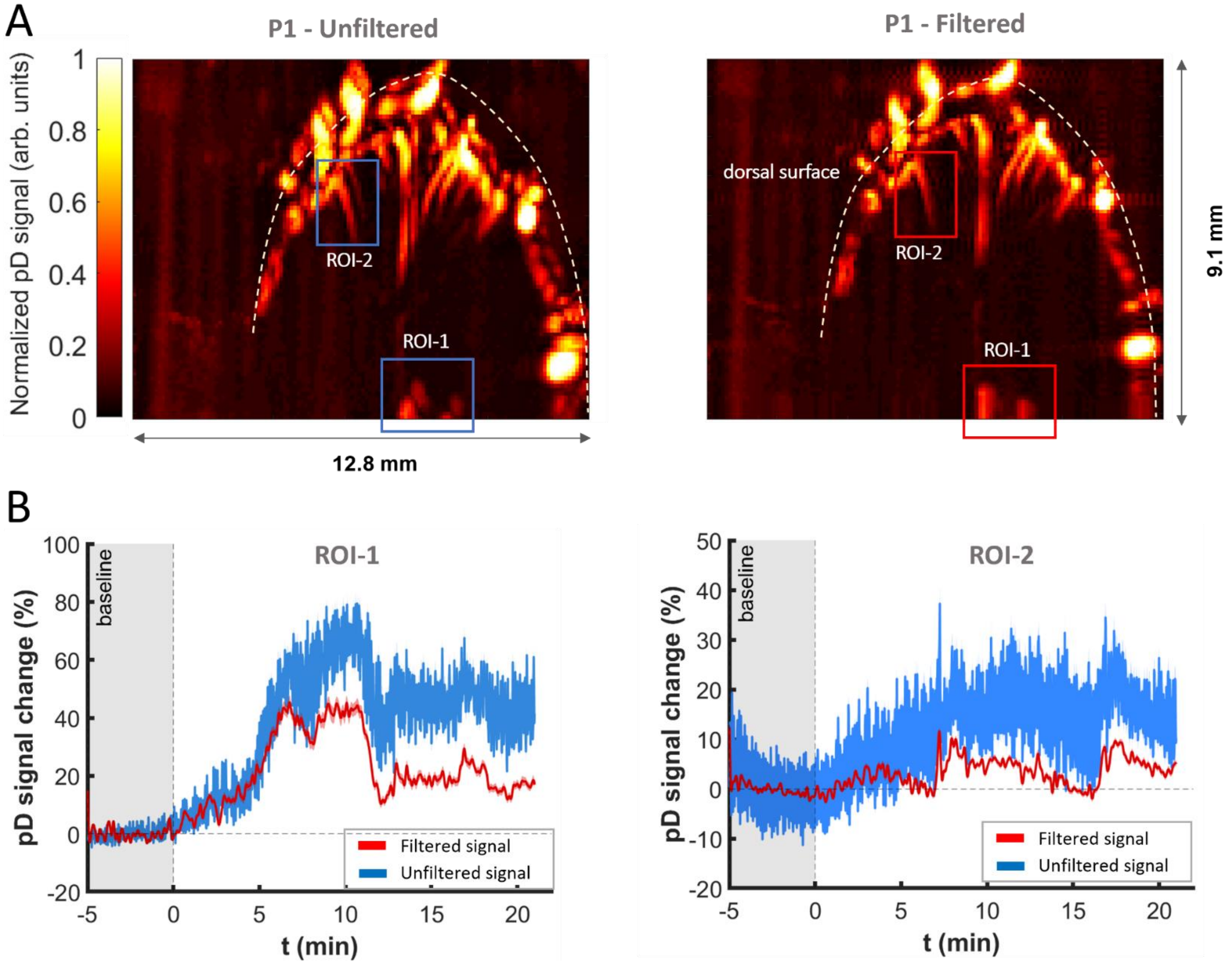

Figure S1, related to Fig. 2. Motion and artifact correction of fUSI of the spinal cord. A) Unfiltered (raw) and filtered mean pD spinal cord activity acquired during baseline, before and after pre-processing, respectively (patient P1). Note that the shift in the pD vascular signal in the unfiltered spinal cord image (left panel) of the indicated regions of interest (ROI-1 and ROI-2) is no longer visible after preprocessing (right panel). **B**) Average %ΔSCBV (i.e., %pD) relative to baseline activity in the two regions of interest (ROI-1 and ROI-2) shown in the unfiltered (blue curve) and filtered (red curve) spinal cord vascular map images (panel A). The ΔSCBV waveforms (red curves) illustrate the resulting signal generated from the highlighted regions after motion, drift trends, and high-frequency artifacts are removed. Source data are provided as a Source Data file.

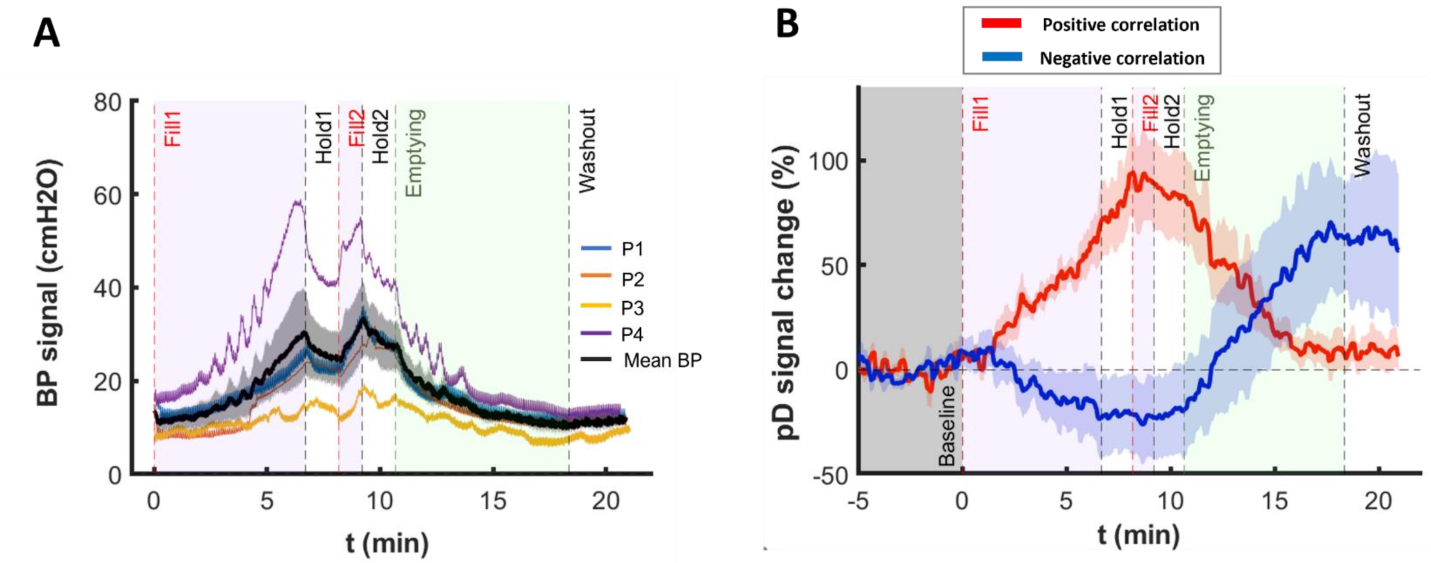

Figure S2, related to Fig. 1 and Fig. 3. A) Non-normalized bladder pressure (BP) signal over time during bladder filling and emptying for the four patients (P1, P2, P3, P4 – blue, orange, yellow and purple, respectively) in the recorded pressure units (cmH2O). The mean BP across all patients is represented by the black curve, with the standard error shaded in gray. **B**) Average % ΔSCBV in the top 5% of significantly activated spinal cord regions during bladder filling and emptying across all patients. The average % ΔSCBV is determined by averaging the %pD signal change over the top 5% significant spinal cord regions that are positively (red curve) and negatively (blue curve) correlated with the bladder pressure. The shaded regions around the ΔSCBV curves represent the standard error, resulting from averaging across all patients. Source data are provided as a Source Data file.

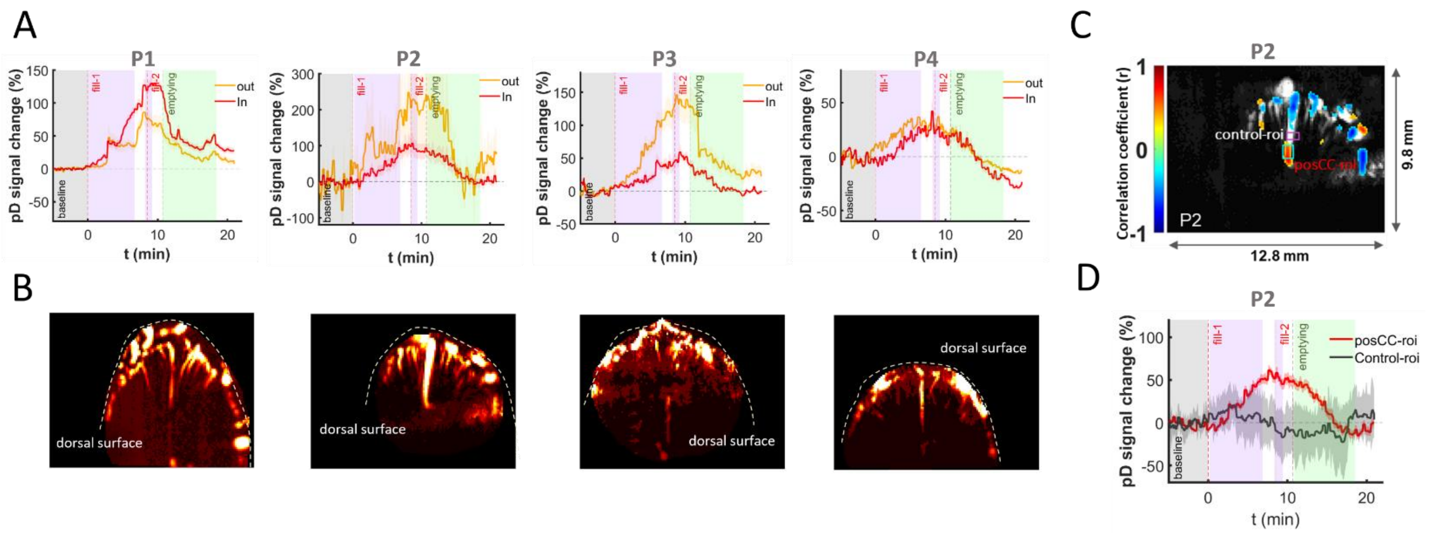

Figure S3, related to Fig. 3. Correlation of regions inside and outside the spinal cord to bladder pressure. **A**) Average % ΔSCBV in significantly activated regions within (red curve) and outside (orange curve) the dorsal surface of the spinal cord that are positively correlated with the bladder pressure during bladder filling and emptying for each patient. The ΔSCBV waveforms exhibit similar increasing and decreasing responses during bladder filling and emptying, both inside and outside the dorsal surface of the spinal cord across all patients. Note that the ΔSCBV curves were generated using the top 5% of regions with significant positive correlations to bladder pressure, both within and outside the dorsal surface of the spinal cord. The shaded regions around the ΔSCBV curves represent the standard error, calculated from averaging across the top 5% of significant regions. **B**) pD vascular maps depicting the designated regions within the dorsal surface of the spinal cord for the four patients. **C**) Activation map of spinal cord regions from patient 2 (P2) with significant positive and negative correlations to bladder pressure, from which a positively activated (posCC-roi) region and a control non-activated (control-roi) region were selected to compare the ΔSCBV waveforms in panel D. **D**) Average % ΔSCBV (i.e., % pD signal change) curves derived from the highlighted ROIs in panel C. The ΔSCBV waveform of the control ROI (black curve) mostly remains flat, uncorrelated with the BP and does not exhibit the increasing and decreasing responses during bladder filling and emptying, observed for the ΔSCBV waveform of the activated ROI (red curve). The shaded regions around the posCC-roi and the control-roi ΔSCBV curves represent the standard error resulting from averaging activity across the selected regions. Source data are provided as a Source Data file.

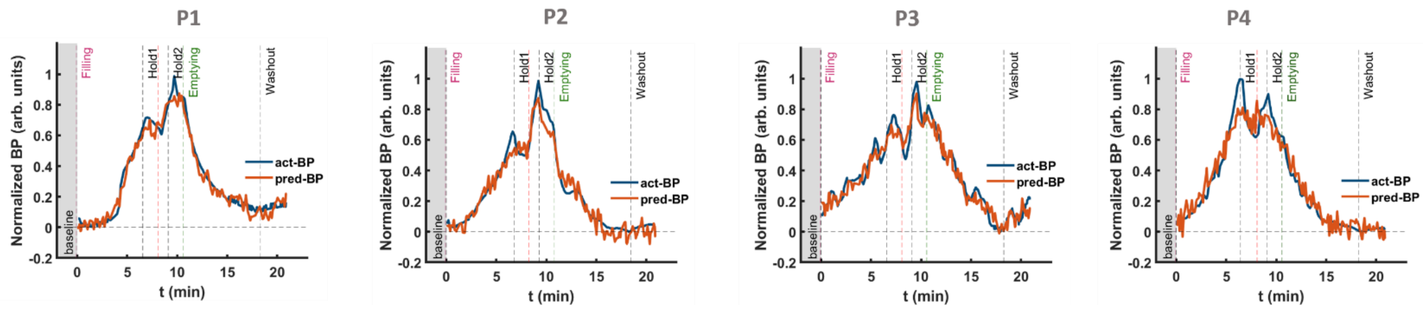

Figure S4, related to Fig. 5. Bladder pressure reconstruction using SVM-r. Actual (act-BP, blue curve) and SVM-r algorithm predicted (pred-BP, orange curve) bladder pressure for the 4 patients. Source data are provided as a Source Data file.
