# Supplementary material for: Human spinal cord activation during filling and emptying of the bladder
Source: Nat Commun. 2025 Jul 15;16:6506. doi: 10.1038/s41467-025-61470-1 (PMC12264007; doi:10.1038/s41467-025-61470-1)
Supplement: Supplementary file 2 — Description of Additional Supplementary Files [file 41467_2025_61470_MOESM2_ESM.pdf]

Description of Additional Supplementary Files:

Supplementary Movie 1: related to Fig. 2. Raw pD images. Raw (unfiltered) pD spinal cord activity recorded from Patient 4 (P4), spanning the 5-minute baseline period followed by the first bladder filling phase, for a total duration of 11 minutes and 40 seconds. Motion artifacts are evident in the unfiltered pD signal – highlighted, for example, by the cyan arrow (displayed at 20 frames per second for visualization).

Supplementary Movie 2: related to Fig. 2. Motion-corrected pD images. Postprocessed (filtered) pD spinal cord activity after applying motion correction and artifact removal. The filtered video shows a stabilized pD signal with motion-induced fluctuations effectively eliminated. Notably, the mean standard deviation of the pD signal across all pixels decreased following filtering, from 10.88 ± 0.43 arb. units (Mean ± SE) in the raw data to 6.34 ± 0.25 arb. units in the filtered data, measured over the entire duration of the recordings
