# Supplementary material for: Human spinal cord activation during filling and emptying of the bladder
Source: Nat Commun. 2025 Jul 15;16:6506. doi: 10.1038/s41467-025-61470-1 (PMC12264007; doi:10.1038/s41467-025-61470-1)
Supplement: Supplementary file 5 — Reporting Summary [file 41467_2025_61470_MOESM5_ESM.pdf]

nature portfolio

Corresponding author(s): Charles Liu, Vassilios N. Christopoulos

Last updated by author(s): Jun 3, 2025

Reporting Summary

Nature Portfolio wishes to improve the reproducibility of the work that we publish. This form provides structure for consistency and transparency in reporting. For further information on Nature Portfolio policies, see our Editorial Policies and the Editorial Policy Checklist.

Statistics

For all statistical analyses, confirm that the following items are present in the figure legend, table legend, main text, or Methods section.

n/a	Confirmed	
☐	☒	The exact sample size (n) for each experimental group/condition, given as a discrete number and unit of measurement
☒	☐	A statement on whether measurements were taken from distinct samples or whether the same sample was measured repeatedly
☐	☒	The statistical test(s) used AND whether they are one- or two-sided<br>*Only common tests should be described solely by name; describe more complex techniques in the Methods section.*
☒	☐	A description of all covariates tested
☐	☒	A description of any assumptions or corrections, such as tests of normality and adjustment for multiple comparisons
☐	☒	A full description of the statistical parameters including central tendency (e.g. means) or other basic estimates (e.g. regression coefficient) AND variation (e.g. standard deviation) or associated estimates of uncertainty (e.g. confidence intervals)
☐	☒	For null hypothesis testing, the test statistic (e.g. F, t, r) with confidence intervals, effect sizes, degrees of freedom and P value noted<br>*Give P values as exact values whenever suitable.*
☒	☐	For Bayesian analysis, information on the choice of priors and Markov chain Monte Carlo settings
☒	☐	For hierarchical and complex designs, identification of the appropriate level for tests and full reporting of outcomes
☐	☒	Estimates of effect sizes (e.g. Cohen's d, Pearson's r), indicating how they were calculated

Our web collection on statistics for biologists contains articles on many of the points above.

Software and code

Policy information about availability of computer code

Data collection	The functional ultrasound imaging data were collected using a build-in software (name IcoScan) of the Iconeus One ultrasound scanner. Since Iconeus One is a commercial scanner, the software for generating the ultrasound sequences and collecting the power Doppler signal is not available to the public.
Data analysis	We used the NoRMCorre algorithm (public available; implemented in Matlab) for rigid motion correction of the raw functional ultrasound imaging data. This algorithm was combined with in-house developed breathing, high frequency smoothing filtering and image stabilization approaches, to ensure robustness and reliability of our data processing and analysis results. Pre-processing, statistical analysis and machine learning analysis performed using Matlab 2024Ra.

For manuscripts utilizing custom algorithms or software that are central to the research but not yet described in published literature, software must be made available to editors and reviewers. We strongly encourage code deposition in a community repository (e.g. GitHub). See the Nature Portfolio guidelines for submitting code & software for further information.

Data

Policy information about availability of data

All manuscripts must include a data availability statement. This statement should provide the following information, where applicable:

- Accession codes, unique identifiers, or web links for publicly available datasets
- A description of any restrictions on data availability
- For clinical datasets or third party data, please ensure that the statement adheres to our policy

Provide your data availability statement here.

Research involving human participants, their data, or biological material

Policy information about studies with human participants or human data. See also policy information about sex, gender (identity/presentation), and sexual orientation and race, ethnicity and racism.

Reporting on sex and gender	Our patients were not specifically recruited or selected to be included in this study. Instead, they were planned for a standard of care spinal stimulator trial surgery. Thus there is no pre-set distribution of sex/gender, race and ethnicity of patients included in the study. Patient inclusion is not limited to any specific group based on sex/gender, race, ethnicity or any other such parameter.
Reporting on race, ethnicity, or other socially relevant groupings	See above.
Population characteristics	No testing for differences in effect or outcome among sex/gender, racial and/ or ethnic groups is performed in the proposed study as it is not within the scope of this study.
Recruitment	Recruitment plan:<br><br>Patients must be between 18 and 85 years and diagnosed with failed back syndrome or chronic back pain. Any patient who meets this inclusion criteria and has signed informed consent, is deemed eligible and is included in the study. Patients were recruited through neurosurgery and pain management clinic visits. No specific recruitment activities were performed for inclusion in the proposed study.
Ethics oversight	All experimental procedures were approved by the University of Southern California (USC) Institutional Review Board.

Note that full information on the approval of the study protocol must also be provided in the manuscript.

Field-specific reporting

Please select the one below that is the best fit for your research. If you are not sure, read the appropriate sections before making your selection.

[x] Life sciences [ ] Behavioural & social sciences [ ] Ecological, evolutionary & environmental sciences

For a reference copy of the document with all sections, see nature.com/documents/nr-reporting-summary-flat.pdf

Life sciences study design

All studies must disclose on these points even when the disclosure is negative.

Sample size	We determined the number of trials that we need to achieve statistically significant results based on the fact that functional ultrasound imaging can detect 2% hemodynamic changes between different conditions (i.e., filling vs. emptying the bladder). We estimated the total number of trials by performing power analysis. Note that number of trials is balanced to maximize data collection in each patient to maximize power while respecting intraoperative time constraints.
Data exclusions	No data were excluded from this analysis.
Replication	We collected data from more than one patient by performing exactly the same experimental protocol - i.e., filling and emptying the bladder under the same experimental conditions. We also performed exactly the same analysis - i.e., statistical parametric mapping, hemodynamic curves, statistical analysis, and the machine-learning technique. We report no findings that cannot be replicated.
Randomization	Randomization is not applied in our study since the block-design of the urodynamic study can be performed only in one way - i.e., first filling and the emptying the bladder.
Blinding	Blinding is not relevant to our study since we did not perform randomized controlled trials (RCT). However, the investigators that analyze the functional neuroimaging recordings received de-identified data - i.e., they were not aware of the gender/age/name and other demographic data of the patients.

Reporting for specific materials, systems and methods

We require information from authors about some types of materials, experimental systems and methods used in many studies. Here, indicate whether each material, system or method listed is relevant to your study. If you are not sure if a list item applies to your research, read the appropriate section before selecting a response.

Materials & experimental systems

n/a	Involved in the study
☒	☐ Antibodies
☒	☐ Eukaryotic cell lines
☒	☐ Palaeontology and archaeology
☒	☐ Animals and other organisms
☒	☐ Clinical data
☒	☐ Dual use research of concern
☒	☐ Plants

Methods

n/a	Involved in the study
☒	☐ ChIP-seq
☒	☐ Flow cytometry
☒	☐ MRI-based neuroimaging

Plants

Seed stocks

Report on the source of all seed stocks or other plant material used. If applicable, state the seed stock centre and catalogue number. If plant specimens were collected from the field, describe the collection location, date and sampling procedures.

Novel plant genotypes

Describe the methods by which all novel plant genotypes were produced. This includes those generated by transgenic approaches, gene editing, chemical/radiation-based mutagenesis and hybridization. For transgenic lines, describe the transformation method, the number of independent lines analyzed and the generation upon which experiments were performed. For gene-edited lines, describe the editor used, the endogenous sequence targeted for editing, the targeting guide RNA sequence (if applicable) and how the editor was applied.

Authentication

Describe any authentication procedures for each seed stock used or novel genotype generated. Describe any experiments used to assess the effect of a mutation and, where applicable, how potential secondary effects (e.g. second site T-DNA insertions, mosiacism, off-target gene editing) were examined.
